# Phenotypic Characterization and Transcriptomic Profiling of *Ziziphus jujuba* var. *spinosa* Seedlings Under ATP Treatment and Identification of a Novel Lupeol Synthase

**DOI:** 10.3390/plants15213367

**Published:** 2026-10-30

**Authors:** Yansheng Wu, Shenxiu Du, Hualiang Liu, Yimian Ma, Wenjing Wang, Yelin Tian, Chunnian He, Xiangdong Chen, Jianyuan Li, Huan Li

**Affiliations:** 1 Hebei Xingzaoren Utilization Technology Innovation Center, Xingtai 054001, China; 200510640@xttc.edu.cn (Y.W.); hlliu2016@126.com (H.L.); 201920666@xttc.edu.cn (J.L.); 201720665@xttc.edu.cn (H.L.); 2 College of Chemical Engineering and Biotechnology, Xingtai University, Xingtai 054001, China; 3 Biotechnology Research Institute, Chinese Academy of Agricultural Sciences, Beijing 100193, China; dushenxiu@caas.cn; 4 National Engineering Laboratory for Breeding of Endangered Medicinal Materials, Beijing 100193, China; 5 Institute of Medicinal Plant Development, Chinese Academy of Medical Sciences & Peking Union Medical College, Beijing 100193, China; cnhe@implad.ac.cn (C.H.); xdchen@implad.ac.cn (X.C.); 6 School of Landscape Architecture, Beijing University of Agriculture, Beijing 102206, China; wwj15246226190@163.com (W.W.); tianyelin@126.com (Y.T.)

**Keywords:** sour jujube, adenosine triphosphate, triterpenoid, mevalonic acid pathway, oxidosqualene cyclase, lupeol synthase

## Abstract

The tender leaves of *Ziziphus jujuba* var. *spinosa* (sour jujube) are widely consumed in China as a health-promoting tea, with triterpenoids being identified as one of their core bioactive constituents. However, key genes in the triterpenoid biosynthesis pathway remain largely uncharacterized in this species. Here, adenosine triphosphate (ATP) treatment inhibited sour jujube seedling growth, induced leaf curling and marginal withering in partial seedlings, and elevated triterpenoid content in seedling aboveground tissues. Transcriptome analysis showed that most mevalonic acid (MVA) pathway enzyme genes were not significantly altered, while some were downregulated, and some enzyme isoforms were slightly upregulated. Combined genome and transcriptome analysis identified one slightly upregulated oxidosqualene cyclase (OSC) gene, *ZsLUS*, among 13 sour jujube OSC family genes. Heterologous expression in yeast confirmed that *ZsLUS* encodes a bona fide lupeol synthase with catalytic activity. These results indicate that ATP treatment enhances triterpenoid accumulation without uniformly upregulating MVA pathway genes, suggesting a sub-pathway-specific regulatory mode or post-transcriptional regulatory mechanism for triterpenoid biosynthesis in sour jujube seedlings. This work deepens our understanding of the mechanism of ATP action on sour jujube triterpenoid biosynthesis and provides new clues for deciphering triterpenoid biosynthesis and accumulation in sour jujube leaves.

## 1. Introduction

*Ziziphus jujuba* Mill. var. *spinosa*, a perennial shrub of the Rhamnaceae family, is considered the ancestor of Chinese jujube (*Z*. *jujuba* Mill.) [1,2]. In China, it is commonly known as sour jujube because of the sour taste of its fruits. The dried seeds of the sour jujube tree, known as Ziziphi Spinosae Semen or Sunzaoren, are widely used in traditional Chinese medicine for their sedative and hypnotic effects, with triterpenoids as the major active ingredients [3,4,5]. Beyond the seeds, the young leaves of the sour jujube tree also possess medicinal value. In China, green tea made from these leaves has gained popularity for its notable sedative-hypnotic effects. Research indicates that triterpenoids and flavonoids are the major bioactive compounds in sour jujube leaves [6,7]. However, the biosynthesis pathway of triterpenoids in sour jujube remains largely unknown. Additionally, the influence of environmental factors on the development of sour jujube leaves and their triterpenoid content is still unclear.

Adenosine triphosphate (ATP) is best known as an intracellular energy carrier. However, in 1929, scientists discovered ATP outside mammalian heart cells, suggesting that extracellular ATP (eATP) might have physiological functions [8]. Subsequent studies revealed that ATP is present outside both plant and animal cells, and key components of the eATP pathway were identified [9,10,11,12,13,14]. Research has demonstrated that eATP plays a central role in responding to external stress stimuli [15]. Moreover, eATP is regarded as a multifaceted regulator of plant fitness, participating in processes such as cell death, stomatal movement, and defence responses [16,17,18,19,20,21]. Notably, recent work by Cho et al. showed that the eATP receptor P2K1 can phosphorylate mevalonate kinase (MVK) in response to eATP, thereby altering the cellular metabolism of Arabidopsis [22].

To investigate how stress signalling modulates triterpenoid biosynthesis in sour jujube leaves, an ATP solution was used as a stress signal to treat sour jujube seedlings. By integrating phenotype tracking, triterpenoid quantification, transcriptome profiling and key enzyme gene identification, whether ATP can trigger seedling stress responses and specifically upregulate core enzyme genes in the triterpenoid biosynthesis pathway was tested. The subsequent sections systematically present the physiological, transcriptomic, and chemical analysis datasets, and the gene identification results, and finally discuss the potential functional mechanism underlying ATP-induced triterpenoid biosynthesis and accumulation in sour jujube seedlings.

## 2. Results

### 2.1. The Phenotypes of Sour Jujube Seedlings After ATP Treatment

**Figure 1:**
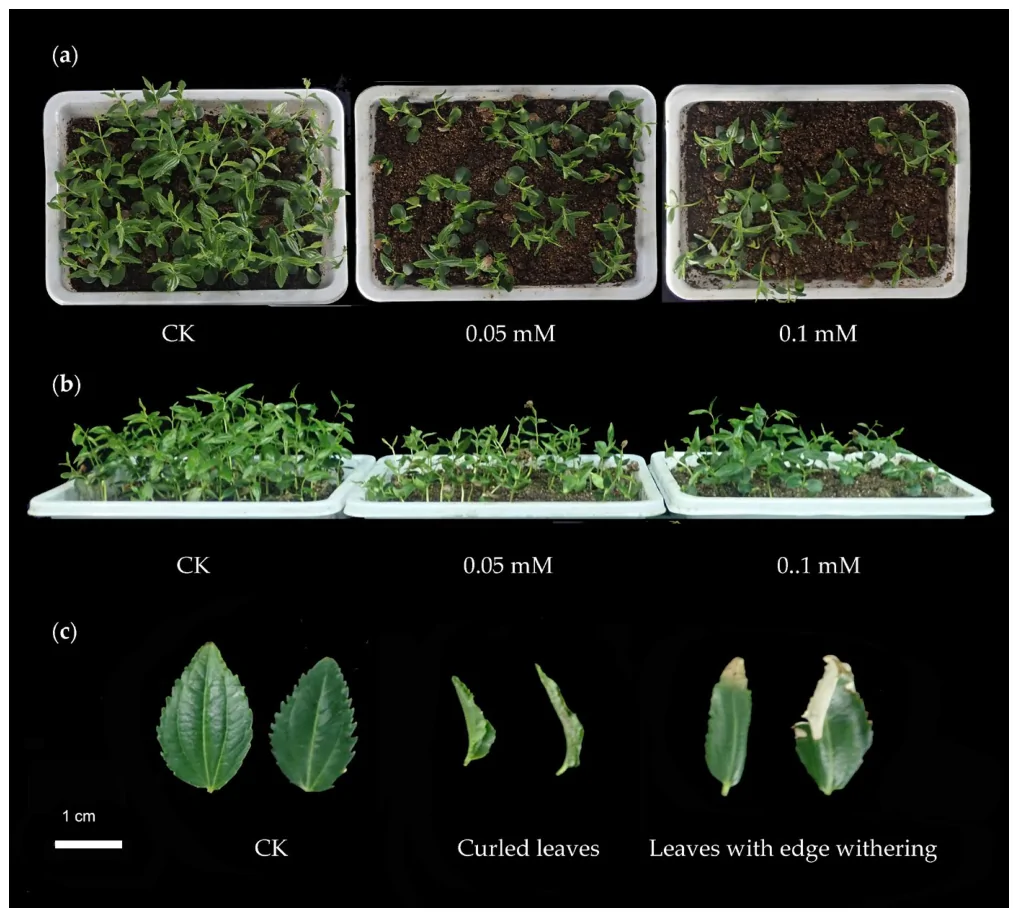
The phenotypes of sour jujube seedlings after four applications of ATP treatment. (**a**) Representative top-view photographs of sour jujube seedling pots 21 days after the first spraying, showing the blank control (CK), 0.05 mM ATP treatment, and 0.1 mM ATP treatment groups. (**b**) Representative side-view photographs of sour jujube seedling pots 21 days after the first spraying, showing the blank control (CK), 0.05 mM ATP treatment, and 0.1 mM ATP treatment groups. (**c**) Phenotypic changes in the sour jujube leaves after ATP treatment. The white line in panel c denotes a 1 cm scale.

In this study, adenosine 5′-triphosphate disodium salt hydrate (C_10_H_14_N_5_O_13_P_3_Na_2_·3H_2_O) solution (ATP solution) was used to simulate exogenous stress signals, as it is a widely used ATP formulation in laboratory research and pharmaceutical applications. First, sour jujube seeds were sown into a cultivation medium with 30% humidity on 8 February 2023, using 30 rectangular pots with 100 seeds per pot. Sixteen days later, on 26 February 2023, the 30 pots of seedlings developed 3–4 true leaves and were subjected to four consecutive weekly treatments performed on 26 February, 5 March, 12 March, and 19 March, respectively. For each treatment, 10 pots of seedlings were sprayed with 0.1 mM ATP solution, another 10 pots with 0.05 mM ATP solution, and the remaining 10 pots (blank control) with distilled water. The seedling phenotype was observed and recorded on 8 March, 13 March, and 18 March 2023. After the fourth ATP treatment, on 20 March, photographs were captured to record seedling phenotypes. As the results show, the ATP-treated seedlings exhibited obvious differences compared to the control, including reduced plant height, fewer true leaves, and increased curled leaves (Figure 1). Notably, the edges of some seedling leaves treated with 0.1 mM ATP were withering (Figure 1c).

**Figure 2:**
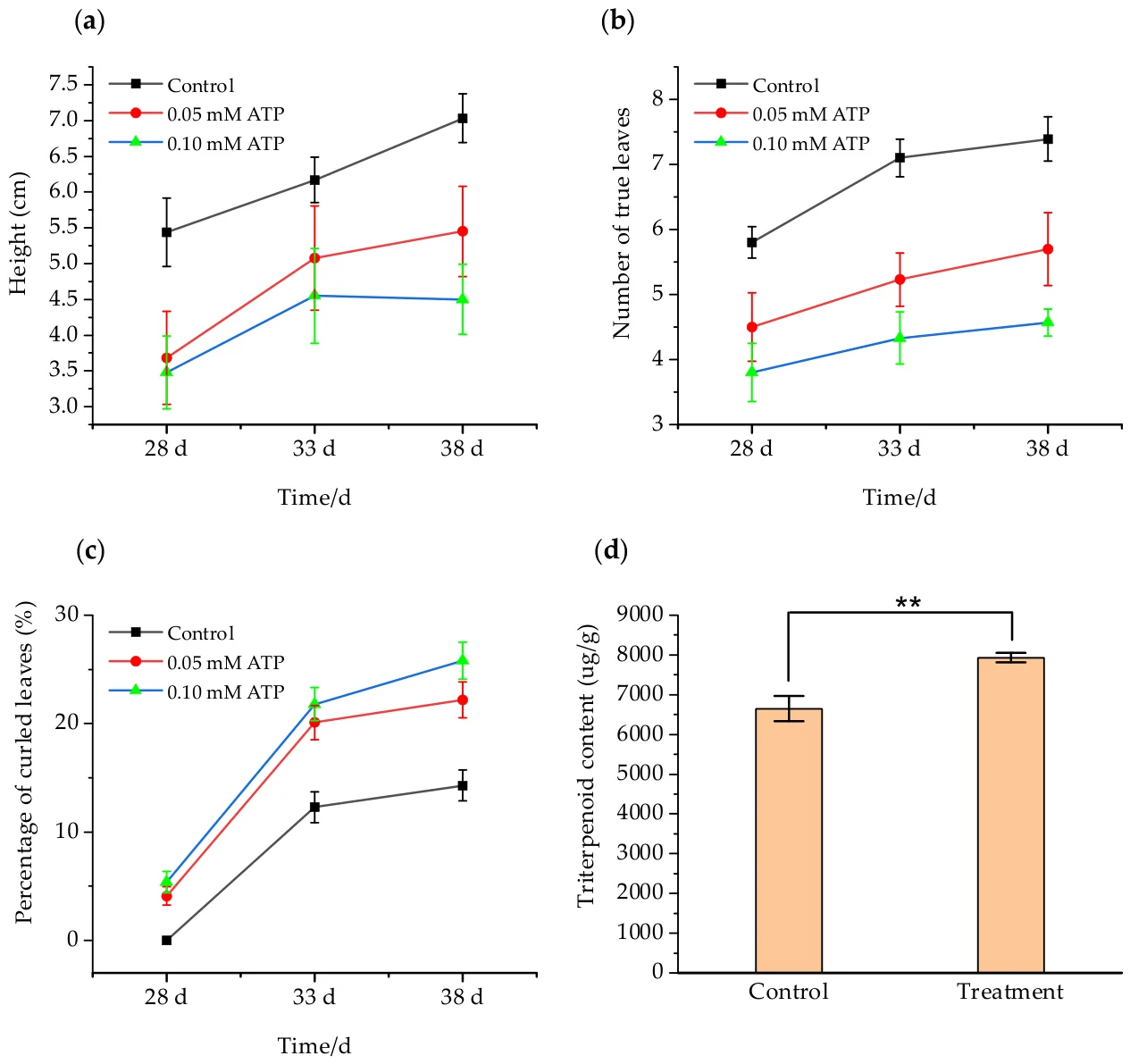
Phenotypic changes and triterpenoid content of sour jujube seedlings after ATP treatment. (**a**) Height changes in the sour jujube seedlings after ATP treatment; (**b**) Changes in the number of true leaves after ATP treatment; (**c**) Changes in the percentage of curled leaves after ATP treatment; (**d**) The effect of 0.1 mM ATP treatment for four times on the total triterpenoid content of sour jujube seedlings. In (**a**–**c**), values represent the mean of 10 biological replicates, and the error bars represent the Mean Absolute Deviation (MAD); while in (**d**), the values represent the mean of three biological replicates, with error bars indicating ± standard deviation (SD). Specifically, ** corresponds to a *p*-value in the range of 0.001 to 0.01, derived from an unpaired two-tailed Student’s *t*-test.

Seedling phenotypic traits, including true leaf number, plant height, and curled leaf percentage, were recorded at 28, 33, and 38 days after sowing, corresponding to 8, 13, and 18 March 2023. ATP treatment reduced seedling height and the number of true leaves, while increasing the percentage of curled leaves (Figure 2). Additionally, after four ATP treatments, on 21 March 2023, total triterpenoid content in the control and 0.1 mM ATP treatment samples was analysed using the Vanillin-HClO_4_ colorimetric method. The assay used oleanolic acid as the standard to estimate total triterpenoid content in the aboveground tissues of sour jujube seedlings. As shown in Figure 2d, the total triterpenoid content of the aboveground tissues of 0.1 mM ATP-treated sour jujube seedlings was significantly higher than that of the control group. With equal variances verified by an F-test (*p* = 0.26), an unpaired two-tailed Student’s *t*-test showed the treatment group (7933) had a significantly higher indicator value than the control group (6648, t = 6.54, df = 4, *p* = 0.0028). The ATP treatment group showed a relative percentage increase compared to the control group of nearly 20%. All raw phenotypic records and triterpenoid quantification data are available in Table S1.

### 2.2. Transcriptomic High-Throughput Sequencing and Data Analysis

**Figure 3:**
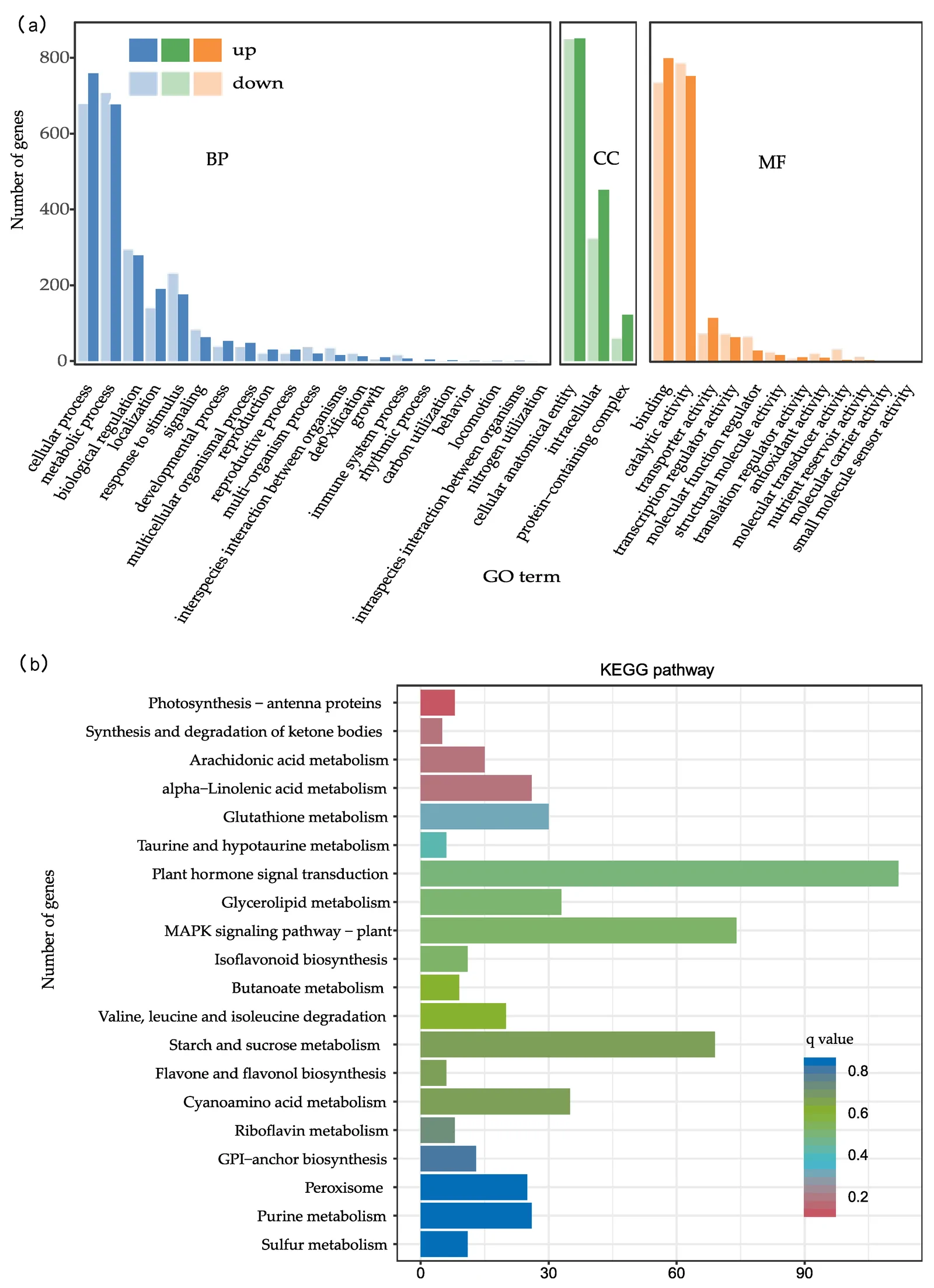
Gene classification results using the GO and KEGG databases. (**a**) The number of genes classified in GO terms. BP, CC, and MF represent biological process, cellular component, and molecular function, respectively. (**b**) The number of genes in the top 20 KEGG pathways with the smallest q-values (highest enrichment significance).

After four weekly treatments with 0.1 mM ATP solution, the aboveground tissues of both control and 0.1 mM ATP-treated seedlings were collected for RNA sequencing on 31 March 2023. The samples were labelled as yc (the aboveground tissues of the untreated control) and yt01 (the aboveground tissues of the seedlings treated with 0.1 mM ATP solution), with three biological replicates per group. RNA Sequencing was performed on an Illumina Novaseq X platform using a standard analysis pipeline. Upon completion of the analysis for all samples, a total of 39.7 Gb of data was obtained. After sequence filtration, the clean data were acquired. The resulting clean data for each sample reached 5.81 Gb or higher, and the Q30 base percentage was 95.47% or higher. Next, clean reads were aligned with the *Z. jujuba* var. *spinosa* reference genome (v. GCA_020796205.1) using the HISAT2 software and new genes were predicted using StringTie [23,24]. The expression levels of all genes were measured (Table S2). A total of 3977 genes were identified as significantly differentially expressed genes (DEGs) using the DEGseq2 software [25]. Among these, 1895 genes were upregulated in the leaves following ATP treatment, while 2082 genes were downregulated. Bioinformatics database searches annotated 3844 of these genes (Table S3). Through searching the GO database, within “biological process” class, “cellular process” and “metabolic process” harboured the largest number of differentially expressed genes; within the “cellular component” class, “cellular anatomical entity” and “intracellular” harboured the largest number of differentially expressed genes; within the “molecular function” class, “binding” and “catalytic activity” represented the two most highly represented subcategories, harbouring the largest number of differentially expressed genes (Figure 3a). The KEGG pathway enrichment analysis showed that the most enriched pathway was “photosynthesis—antenna proteins”. Although the ‘Plant hormone signal transduction’ and ‘MAPK signalling’ pathways contained a relatively high number of differentially expressed genes, their enrichment did not reach statistical significance after multiple-testing correction (*p* < 0.05). Therefore, they are not discussed as primary enriched pathways in this study (Figure 3b).

### 2.3. Identification of the Differentially Expressed Enzyme Genes in the Triterpenoid Biosynthesis Pathway

The 2-C-methyl-D-erythritol 4-phosphate (MEP) pathway and the MVA pathway are two key metabolic pathways responsible for the synthesis of isoprene pyrophosphate (IPP) and dimethylallyl pyrophosphate (DMAPP), which are precursors of terpenoids. To characterize gene expression changes in the triterpenoid biosynthesis pathway after ATP treatment, we mapped the significantly differentially expressed genes (DEGs) to the KEGG pathway map KO00900 (Terpenoid backbone biosynthesis). DEGs were identified with the thresholds of $|\log_{2}\left(FoldChange\right)|$ ≥ 1 and FDR (False Discovery Rate) < 0.01. Analysis of DEGs in the KO00900 map (Terpenoid backbone biosynthesis) showed that after four weekly doses of ATP treatment, most genes in the conserved MEP and MVA pathways were not significantly upregulated, while some were even downregulated. This result showed that the expression of most enzyme-encoding genes in the MVA pathway remained normal or was significantly downregulated in ATP-treated sour jujube seedlings, when evaluated against a strict criterion. However, when comparing the average FPKM values, several gene isoforms exhibited slightly elevated expression levels (Table S4). Specifically, the expression levels of enzyme genes encoding MVK and PMK were increased by approximately 2-fold, though with relatively low basal expression levels. These two enzymes are the rate-limiting enzymes of the MVA pathway, and thus these increases likely have specific biological implications. To systematically dissect the ATP-induced transcriptional changes in triterpenoid biosynthesis, we performed an in-depth expression profiling analysis on the full set of mid- and downstream enzyme-encoding genes in the triterpenoid biosynthetic pathway. Following the MVA and MEP pathways, the conserved linear catalytic sequence for triterpenoid skeleton biosynthesis proceeds as follows: the C5 isoprenoid precursors IPP and DMAPP are polymerized into farnesyl pyrophosphate (FPP) by farnesyl pyrophosphate synthase (FPPS). FPP is then converted to squalene by the action of squalene synthase (SQS), which is further epoxidized by squalene epoxidase (SQE) to form 2,3-oxidosqualene. This key intermediate is cyclized by distinct oxidosqualene cyclases (OSCs) to produce various triterpenoid skeletons. The DEG analysis results showed that different isoforms of enzyme genes also showed distinct expression patterns (Table S4).

In sour jujube, OSCs are enzymes responsible for the formation of triterpenoid skeletons, such as lupeol, α-amyrin, and β-amyrin, which will convert the major triterpenoids in sour jujube, such as betulinic acid, ursolic acid, and oleanolic acid by modifying enzymes. So, OSCs are the first key branch-point enzymes for the formation of sour jujube triterpenoids and determine the triterpenoid content during jujube domestication [26]. To identify genes encoding OSCs, all sour jujube proteins were downloaded from the genome database National Centre of Biotechnology Information (NCBI) (https://www.ncbi.nlm.nih.gov/datasets/genome/GCF_020796205.1/, accessed on 21 March 2025). Using the *Arabidopsis* oxidosqualene cyclase genes AtLUP1 (At1g78970), AtCAS1 (At2g07050), and AtLAS1 (At3g45130) as queries, homologous OSCs from sour jujube proteins were searched using a local BLAST method (NCBI BLAST v. 2.12.0) against the sour jujube proteins [27]. After removing duplicate sequences, protein domains were identified by searching the NCBI Conserved Domain Database. Given that the PLN03012 domain (Camelliol C synthase domain) is a defining feature of OSC family members, the OSCs that lack this domain were removed. Furthermore, because plant OSCs typically range from 490 to 900 amino acids in length, all sour jujube OSC sequences that were shorter than 490 aa or longer than 900 aa were removed. After all, a total of 13 non-redundant OSC proteins were ultimately identified. The OSC proteins were also searched against the NCBI gene database to acquire their gene symbol and locus tag. The basic physicochemical properties of the sour jujube OSC gene family members, such as the theoretical isoelectric point and the molecular weight of proteins, were acquired using the online tool ProtParam (http://web.expasy.org/protparam/, accessed on 1 October 2026). The subcellular localization of these proteins was predicted using the online tool WoLF PSORT (https://wolfpsort.hgc.jp/, accessed on 1 October 2026). The expression changes in the OSC genes were analysed by comparing their FPKM values based on the transcriptome data. Finally, all characteristics of the 13 OSC proteins are shown in Table S5.

### 2.4. Phylogenetic Analysis of the OSC Families and Expression Level Comparison

**Figure 4:**
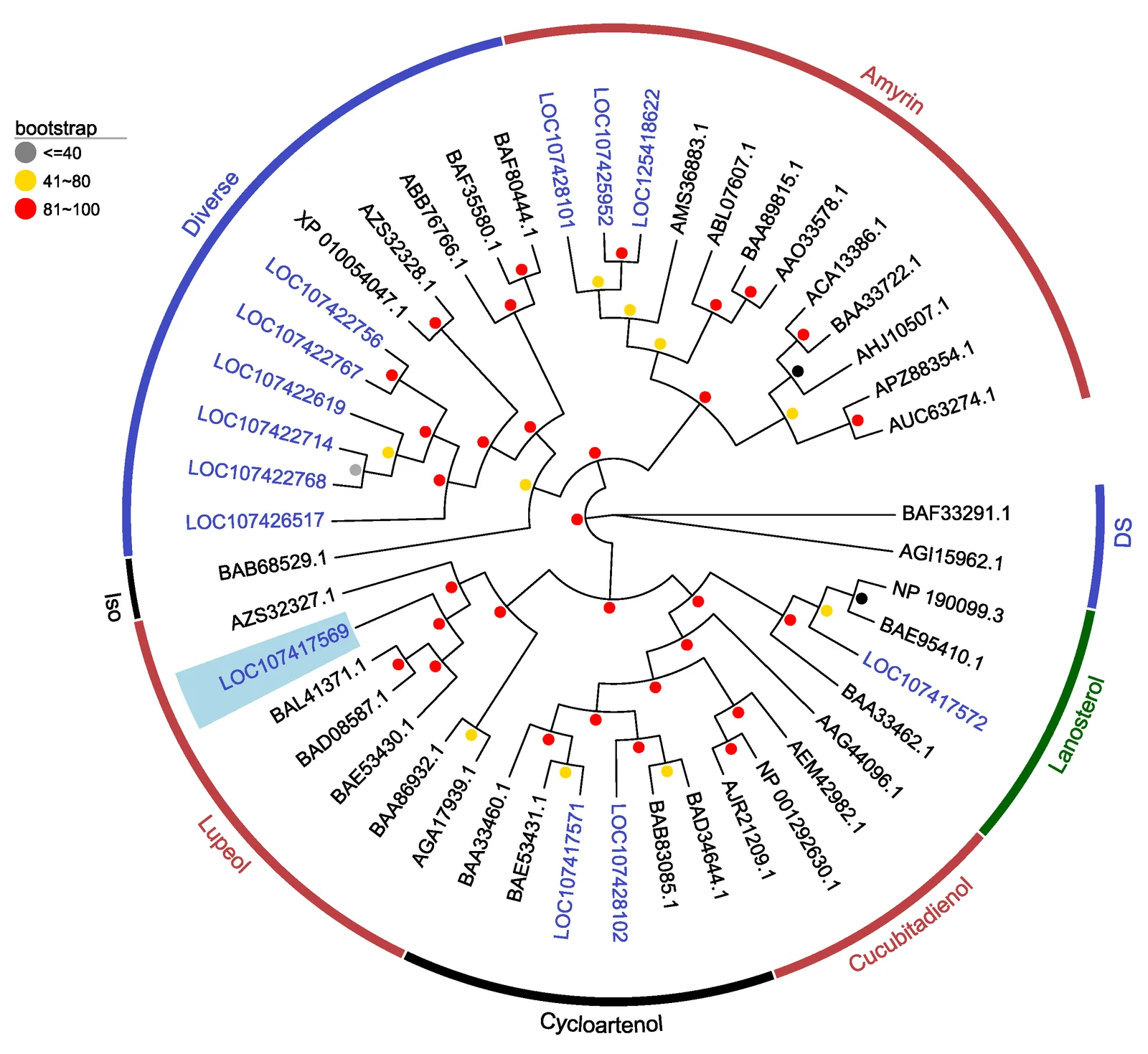
The unrooted phylogenetic tree of ZsOSCs and other OSCs using the maximum likelihood (ML) method, with reliability assessed by a bootstrap test (1000 replicates). Possible enzyme products are provided for each group. The group names “DS” and “Iso” are abbreviations of “Dammarendiol” and “Isomultiflorenol”, respectively. All ZsOSC sequences are represented by their GenBank gene symbols prefixed with “LOC” and highlighted in royal blue, while all other plant OSC entries are represented by their respective GenBank accession numbers. As per the legend, the grey, yellow, and red circles at the nodes represent bootstrap values of 0–40%, 41–80%, and 81–100%, respectively.

To further identify the possible products of sour jujube OSCs, an unrooted maximum likelihood (ML) phylogenetic tree was constructed using the 13 sour jujube OSCs combined with other known plant OSCs. As shown in Figure 4, the OSCs were divided into eight clusters. Three sour jujube OSCs belonged to the amyrin cluster, six to the diverse products cluster, two to the cycloartenol cluster, one to the lupeol cluster, and one to the lanosterol cluster. It was evident that the gene descriptions provided by GenBank were not entirely consistent with the phylogenetic analysis results (Table S5). Therefore, the functions of sour jujube OSC genes require further in-depth characterization.

Based on FPKM value comparisons, the two genes encoding sour jujube OSCs in the cycloartenol cluster (NewGene_975 and FEM48_Zijuj10G0003600) exhibited increased expression levels in the aboveground tissues of sour jujube seedlings after ATP treatment. One gene encoding OSC in the lupeol biosynthesis cluster, originally annotated as LOC107417569 (FEM48_Zijuj05G0005300), was upregulated under exogenous ATP treatment. Its mean FPKM value increased from 38.71 in the control group to 47.51 in the ATP treatment group, representing a 1.23-fold induction. This gene was designated as *ZsLUS*. Among the remaining OSC genes, one with the description of amyrin synthase (NewGene_5814) exhibited relatively high basal expression in sour jujube leaves but showed a downregulation trend after ATP treatment, with mean FPKM values of 24.31 and 3.01 in the two groups. Another gene defined as β-amyrin synthase 1 (NewGene_3451) showed low basal expression in leaves but was significantly upregulated after ATP treatment, with mean FPKM values of 2.36 and 8.13. The protein encoded by this gene clustered into the diverse clade in the phylogenetic analysis. All other sour jujube OSC members maintained extremely low transcript abundance (FPKM value < 1) in the aboveground tissues of sour jujube seedlings and were excluded from the expression analysis.

### 2.5. Validation of the Function of ZsLUS by Heterologous Expression in Yeast

**Figure 5:**
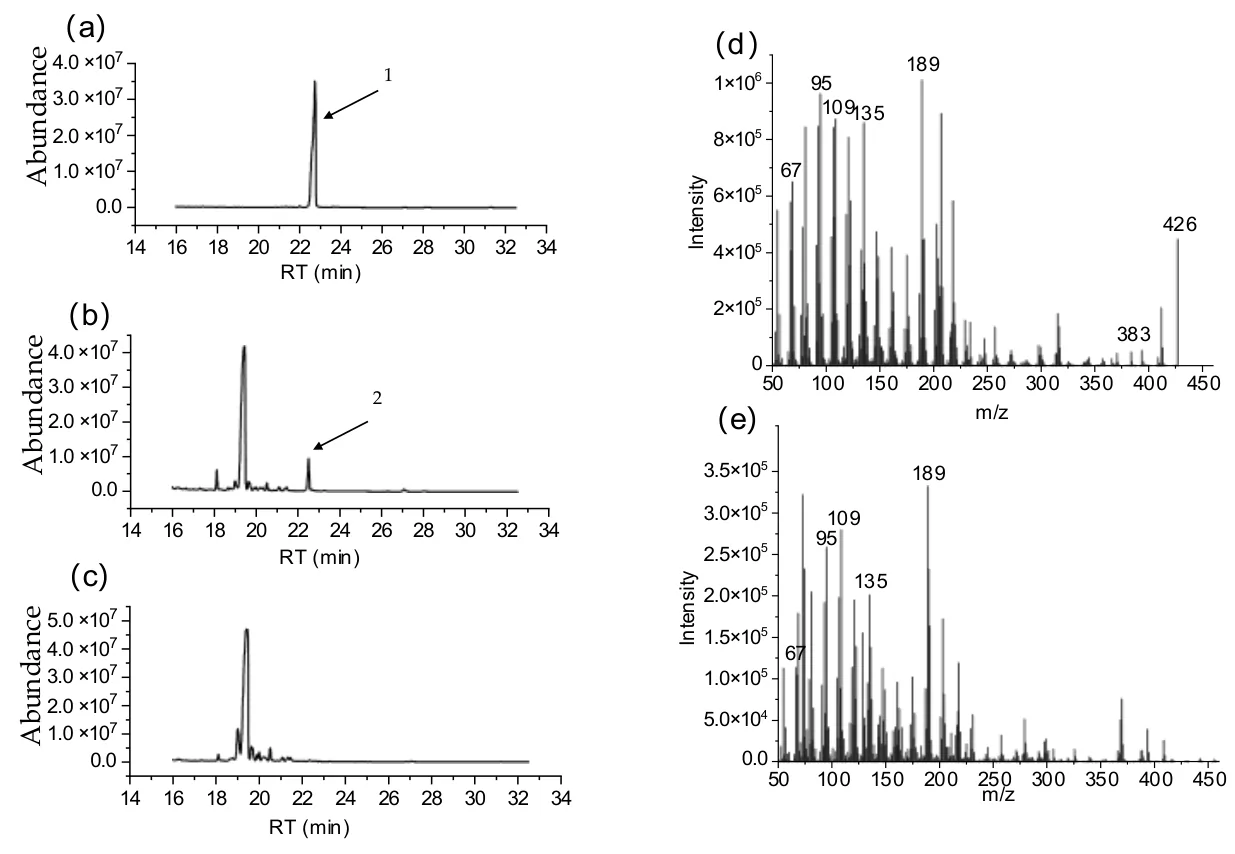
Gas chromatography-mass spectrometry (GC–MS) detection of ZsLUS catalytic reaction products. RT represents the retention time. (**a**) Total ion chromatogram (TIC) of the authentic lupeol standard. (**b**) TIC of the *ZsLUS*-expressing yeast extract. (**c**) TIC of the empty vector control extract. (**d**,**e**) Mass spectra of peaks 1 and 2, respectively. Peak 1 exhibits an RT and mass spectrum identical to those of Peak 2, which corresponds to authentic lupeol, while this peak was not present in the TIC of the empty vector control extract.

Based on the above results, *ZsLUS* was predicted to encode a lupeol synthase. The in vivo function of this gene was investigated. Firstly, this gene was amplified from the young leaves of a two-year-old sour jujube plant. Then, this gene was recombined with a pESC-His vector and transformed into the *Saccharomyces cerevisiae* strain WAT11. After galactose induction, the chemicals produced by the engineered yeast were analysed. A gas chromatography-mass spectrometry (GC-MS) analysis was performed on an Agilent 7890B gas chromatograph coupled to an Agilent 5977A mass selective detector (Agilent Technologies, Santa Clara, CA, USA). Separation was carried out using an HP-5 MS capillary column (30 m × 0.25 mm i.d., 0.25 μm film thickness). Consistent with our prediction, the transgenic yeast cells overexpressing *ZsLUS* produced a new product that was not present in the control yeast cells carrying the empty vector pESC-His (Figure 5). The new compound was identified as lupeol by comparing its retention time and mass spectra to the authentic standard. The result suggested that *ZsLUS* is an enzyme gene involved in the production of lupeol and lupane-type pentacyclic triterpenoids in sour jujube seedlings.

## 3. Discussion

### 3.1. ATP Treatment Improves the Accumulation of Triterpenoids in Sour Jujube Seedlings

In this study, the triterpenoid content in sour jujube seedlings after ATP treatment was analysed. This content was found to be increased after four weekly ATP treatments (Figure 2d). To date, it is known that sour jujube leaves contain multiple classes of pentacyclic triterpenoids with valuable pharmacological properties [28,29]. These pentacyclic triterpenoids can be divided into several groups, such as lupane-, oleanane-, ursane-, and ceanothane-types. Guo et al. characterized fourteen constituents in jujube leaves, including three flavonoids, two saponins, and nine triterpene acids [6]. The main triterpene acids and saponins with relatively high content included zizyphus saponin II, epiceanothic acid, alphitolic acid, betulinic acid, hydroxyursolic acid, ceanothic acid, zizyphus saponin I, zizyberanalic acid, and oleanolic acid. The biosynthesis and induction mechanisms of these triterpenoids remain largely uncharacterized.

Although ATP has been known as both an intracellular energy carrier and an extracellular signal in plant stress perception, the physiological outcome of ATP treatment on triterpenoid metabolic flux and final triterpenoid content in plants has not been documented. Here, we report that repeated weekly foliar application of ATP solution can lead to an increase in triterpenoid content in sour jujube seedlings. To our knowledge, this study was the first to demonstrate that the weekly spraying of ATP solution can increase the triterpenoid content in sour jujube seedlings. This offers a theoretical basis for developing cultivation strategies that enhance triterpenoid accumulation in sour jujube leaves.

### 3.2. eATP Alters the Phenotypes of Sour Jujube Seedlings

eATP is a crucial signalling molecule that regulates stomatal movement. It can promote stomatal opening under optimal conditions in species such as *Arabidopsis thaliana* and *Vicia faba* [30,31]. In this study, three consecutive weekly treatments of sour jujube seedlings with 0.05 mM or 0.1 mM ATP resulted in progressive leaf curling, with incidence increasing over time (Figure 1c). We speculate that eATP treatment may affect stomatal regulation and water loss, potentially contributing to leaf curling. Concurrently, some treated leaves exhibited marginal withering rather than curling. Given that eATP is a known modulator of programmed cell death and pathogen defence [18,32], we hypothesize that the ATP treatment triggered cell death pathways in some of the sour jujube leaves, resulting in the observed necrosis at leaf margins. However, this hypothesis remains unvalidated in the present study, direct cytological and physiological measurements should be performed in the future to confirm this process.

### 3.3. Exogenous ATP Did Not Trigger Significant Transcriptional Upregulation of Most Enzyme Genes in Triterpenoid Biosynthesis Pathway

**Figure 6:**
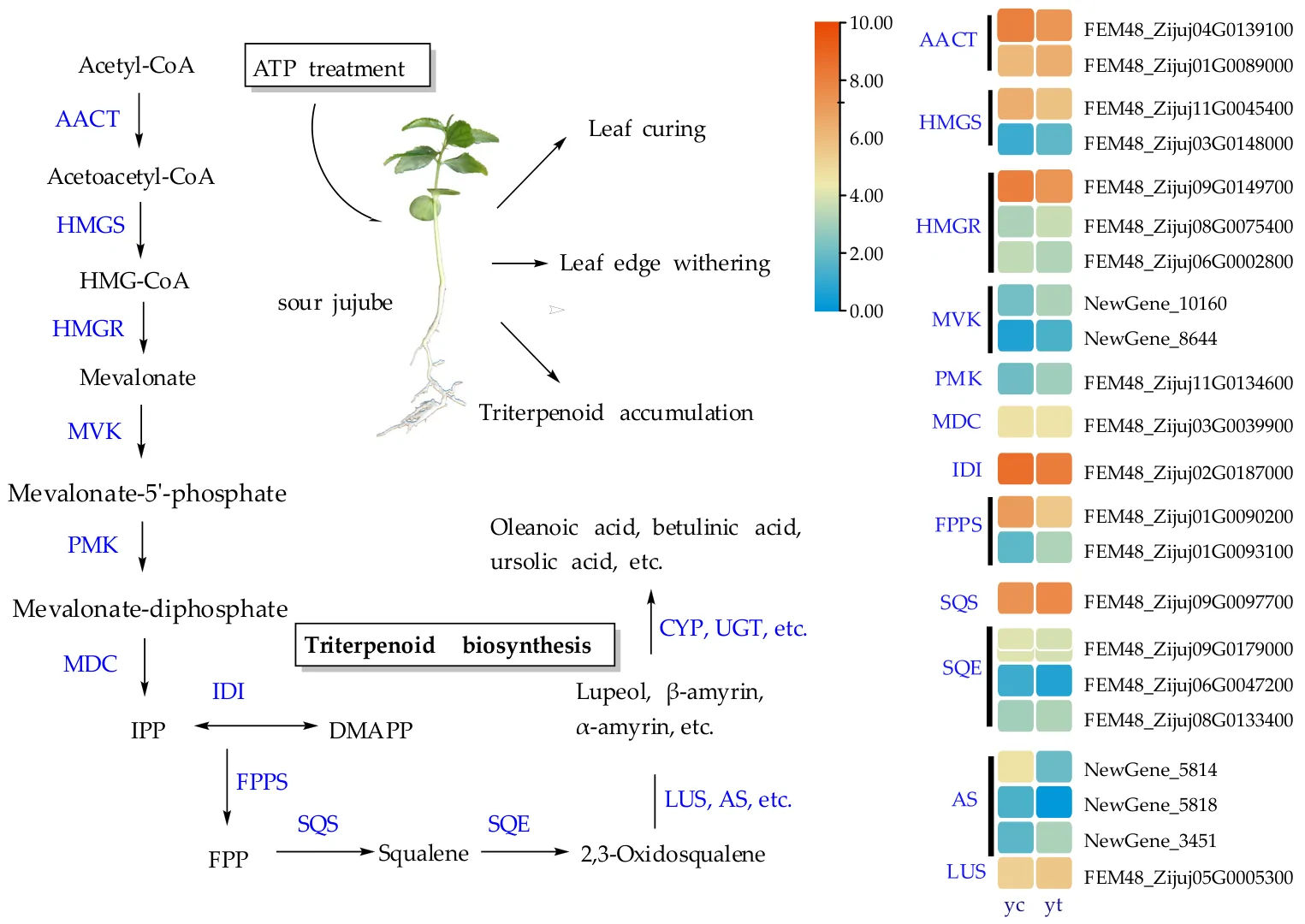
Effects of ATP treatment on the phenotype of sour jujube seedlings and the expression changes in genes involved in the triterpenoid biosynthesis pathway; IPP and DMAPP represent isopentenyl diphosphate and dimethylallyl diphosphate, respectively. Key enzymes also include acetyl-CoA acetyltransferase (AACT), 3-hydroxy-3-methylglutaryl-CoA synthase (HMGS), 3-hydroxy-3-methylglutaryl-CoA reductase (HMGR), mevalonate kinase (MVK), phosphomevalonate kinase (PMK), mevalonate diphosphate decarboxylase (MDC), isopentenyl diphosphate isomerase (IDI), farnesyl diphosphate synthase (FPPS), squalene synthase (SQS), squalene epoxidase (SQE), lupeol synthase (LUS), α-amyrin synthase and β-amyrin synthase (AS), Cytochrome p450 (CYP), and glycosyltransferase (UGT). The expression changes in the enzyme genes were predicted through analysis of transcriptome data and are shown as separate heatmaps; yc and yt represent the control sample and the 0.1 mM ATP-treated sample, respectively. Expression values (average FPKM) are  log2-transformed for heatmap presentation, with rows corresponding to individual genes and columns to samples. For clarity, genes with average expression levels < 1 in all samples were excluded from the heatmap, and CYP and UGT were not included in the analysis.

Phenotypic observations and chemical analyses revealed that repeated treatments with 0.05 mM or 0.1 mM ATP solution stunted the growth of sour jujube seedlings and promoted triterpenoid accumulation. To elucidate the underlying molecular mechanisms, transcriptomic profiling was used to identify DEGs following repeated 0.1 mM ATP treatments. The transcriptomic data indicated that, based on a rigorous evaluation criterion (Fold change > 2 and *p* value < 0.01), the expression levels of most genes encoding MVA pathway enzymes remained normal or were significantly downregulated after ATP treatment. However, when comparing mean FPKM values, some enzyme genes exhibited slightly increased expression levels (Table S4). We also analysed the expression changes of 13 OSC gene family members in sour jujube seedlings after ATP treatment. Based on comparisons of mean FPKM values, the expression of the lupeol synthase gene *ZsLUS* was upregulated, with a relatively higher basal expression level (Table S5). The expression patterns of MVA pathway genes and some representative downstream triterpenoid biosynthetic-related genes are compared in Figure 6.

### 3.4. The Possible Mechanism of ATP-Induced Triterpenoid Biosynthesis and Accumulation

After repeated foliar application of ATP solution, stress-related growth inhibition in sour jujube seedlings occurred, alongside triterpenoid accumulation. Integrated our results and some previous findings, we put forward speculations on the ATP-induced triterpenoid biosynthesis mechanism. In a previous study, Yang et al. have proposed a metabolic strategy in the underground tissues of growth-delayed, two-year-old cultivated *Panax ginseng*. The MVA pathway genes were downregulated to restrict the supply of terpenoid precursors, while branchpoint OSC enzymes were upregulated to divert carbon flux toward defence-associated ginsenosides [33]. In another study, Chi et al. discovered that drought stress induced leaf curling in olives, elevated squalene content in both roots and leaves, and differentially modulated the terpenoid pathway. Most squalene synthesis pathway genes were slightly downregulated, whereas a lupeol synthase gene LUPS, was significantly upregulated in drought-stressed leaves [34]. In the present study, we discovered that ATP treatment induced leaf curling in sour jujube seedlings. The genes involved in the triterpenoid biosynthesis pathway were differentially regulated after ATP treatment. Most triterpenoid pathway genes were downregulated, whereas a partial group of genes was slightly upregulated, including a lupeol synthase gene, *ZsLUS*. Based on the experimental evidence and the existing literature support, we speculate that the upregulation of specific enzyme genes in the triterpenoid biosynthesis pathway may contribute to triterpenoid accumulation in ATP-treated sour jujube seedlings, though most of the MVA pathway genes were not significantly upregulated. Furthermore, a 2022 landmark study demonstrated that the eATP receptor P2K1 can phosphorylate MVK in Arabidopsis in response to eATP, thus activating the MVA pathway and impacting cellular metabolism [22]. Based on this, we speculated that the ATP treatment may mimic eATP signalling, leading to the activation of MVK, a core catalytic component in the MVA pathway, in sour jujube seedlings. The activated MVK would in turn activate a subset of stress-responsive signal molecules and transcription factors, and selectively upregulate stress-related genes, ultimately changing the expression of specific triterpenoid pathway genes and elevating triterpenoid accumulation. However, this is still a preliminary hypothesis, and subsequent experiments are needed to fully validate this proposed regulatory chain. Despite the limitations, this study provides a new clue for understanding stress-induced triterpenoid accumulation in woody medicinal plants. Some of the enzyme genes involved in the triterpenoid biosynthesis characterised in this study may be potential targets to improve the bioactive triterpenoid content in sour jujube and related species.

## 4. Materials and Methods

### 4.1. Plant Materials

The sour jujube seeds used in this study were collected from the same harvest batch of the *Ziziphus jujuba* var. *spinosa* cultivar “Jinxin No. 2” in 2022, and authenticated by Professor Yansheng Wu in Xingtai University. Prior to the experiment, the seeds were first immersed in deionized water, then stored at 4 °C for 3 days for stratification. Afterwards, the seeds were dried at room temperature for approximately 7 days. Then, on 8 February 2023, sour jujube seeds were sown in 30 rectangular plastic flowerpots (inner dimensions: 15.5 cm × 11.4 cm × 5.8 cm) with consistent cultivation medium and drainage conditions. The cultivation medium consisting of perlite and vermiculite at a 1:1 volume ratio with humidity maintained at 30%. The medium humidity was measured using a portable soil environment detector (Waldorf, Hebei, China). In each pot, 100 seeds were evenly sown. All seedlings were cultured in a greenhouse, where the growth conditions were maintained at 22–25 °C with a 12 h light/12 h dark photoperiod. Sixteen days later, when the 30 pots of seedlings developed 3–4 true leaves, they were subjected to four consecutive weekly treatments performed on 26 February, 5 March, 12 March, and 19 March, respectively. For each treatment, ten pots of seedlings were assigned as the blank control group treated with distilled water, ten were subjected to the 0.05 mM ATP solution treatment, and the remaining ten were allocated to the 0.1 mM ATP solution treatment group. The ATP solution was prepared with C_10_H_14_N_5_O_13_P_3_Na_2_·3H_2_O and distilled water and the pH was adjusted to a neutral range. Foliar spraying treatments were performed using 10 mL graduated mini spray bottles, with each pot receiving a 2 mL spray volume per application. The phenotypic traits including plant height, number of true leaves, and percentage of curled leaves were non-invasively monitored at 5-day intervals that began on 8 March 2023. Plant height and number of true leaves were measured, with one individual randomly selected from each pot, resulting in a total of 10 plants for statistical analysis. The percentage of curled leaves was determined by counting the number of curled leaves from approximately 100 leaves, which were randomly sampled from 20 to 25 seedlings per pot. For the height, number of true leaves and curled leaf percentage statistical analysis, Mean Absolute Deviation (MAD) was used to estimate the dispersion of the data for its robustness to outlier values in the small biological replicate dataset. After the fourth treatment (on 30 March 2023), the phenotype of the seedlings was recorded using a digital camera. Then, on the next day, the aboveground tissues of seedlings from both the control and 0.1 mM ATP treatment groups were harvested. One subset of the samples was used for triterpenoid content determination, while the other subset was sent for sequencing.

### 4.2. Testing of Triterpenoid Content

Total triterpenoid content of the samples from the ATP treatment and control groups was determined using a commercial kit (Mreda, Beijing, China) based on the vanillin-HClO_4_ colorimetric method. Briefly, the aboveground tissues of sour jujube seedlings from both control and treatment groups were collected, with three independent biological replicates set up for each group. To form one biological replicate, one seedling was randomly collected from each of 10 independent pots, and the 10 seedlings were pooled together in a tube. This sampling procedure was repeated three times independently to generate three biological replicates. Seedlings with curled leaves were retained in the experiment, while those with withered leaves were excluded. The samples were dried, then pulverized into a fine powder. For each replicate, 0.05 g of sample powder was ultrasonicated in 1 mL 80% ethanol for 1 h. After centrifugation, the supernatant was dried at 70 °C, redissolved in 0.2 mL vanillin-glacial acetic acid and 0.8 mL HClO_4_, and incubated at 55 °C for 20 min. After cooling, 200 μL of the mixture was added to 1 mL glacial acetic acid, and absorbance A_1_ was measured at 589 nm. For the blank control, 0.5 mL 80% ethanol was evaporated to dryness at 70 °C, followed by the same colour reaction procedure. Absorbance A_2_ was recorded under the same conditions, and the final value was calculated as ΔA = A_1_ − A_2_. According to this method, the linear regression equation for the standard oleanolic acid was: y = 0.0072x − 0.012, R^2^ = 0.9968. In this equation, x is the concentration of the standard (μg/mL), and y is ΔA. Therefore, the total triterpenoid content was calculated as: triterpenoid content (μg/g DW) = 138.89 × (ΔA + 0.012) ÷ 0.05, where DW represents dry weight. Standard Deviation (SD) was used to quantify dispersion. Each sample had three technical replicates. Statistical significance between the control and treatment groups was evaluated using the GraphPad Prism software (Version 10.4.1). The equality of variances between the two groups was verified via an F-test (*p* > 0.05), and thus an unpaired two-tailed Student’s *t*-test was subsequently applied for intergroup comparison.

### 4.3. RNA Extraction and Transcriptomic Sequencing

For transcriptome sequencing, the samples were collected using the same sampling procedure as that applied for triterpenoid quantification. Total plant RNA was extracted using the RNAprep Pure Plant Kit (Tiangen, Beijing, China) from the control and treatment groups with three independent biological replicates and following the manufacturer’s instructions. After assessing RNA integrity using the RNA Nano 6000 Assay Kit on the Agilent Bioanalyzer 2100 system (Agilent Technologies, Santa Clara, CA, USA), 1 μg of total RNA per sample was used as input material. Sequencing libraries were generated using the Illumina Hieff NGS Ultima Dual-mode mRNA Library Prep Kit (Yeasen Biotechnology, Shanghai, China). Next, the libraries were sequenced on an Illumina NovaSeq X platform to generate 150 bp paired-end reads.

### 4.4. Bioinformatic Analysis

The raw reads were processed using a bioinformatic pipeline. After removing reads containing adapters, ploy-N, and those of low quality, clean reads were aligned with the *Z. jujuba* var. *spinosa* reference genome (v. GCA_020796205.1) using HISAT2 (v. 2.0.4) [23]. After alignment, the transcripts were assembled using the StringTie (v.2.2.1) software and the annotation result was compared with the original genome annotation information, searching for previously unannotated transcription regions, so as to discover new transcripts and genes [24]. Gene expression levels were estimated by the values of fragments per kilobase of transcript per million fragments mapped (FPKM). Differentially expressed genes were identified using the DEGseq2 software (v. 1.30.1) [25]. The resulting *p* values were adjusted using Benjamini and Hochberg’s approach to control the false discovery rate. DEGs with significance were selected based on the criteria: ${|log2}^{\left(FoldChange\right)}|$ > 1 and padj < 0.01. The functions of significant differentially expressed genes were annotated by searching the following databases: Nr (NCBI Non-Redundant Protein Sequences); Pfam (Protein Family); KOG/COG (Clusters of Orthologous Groups of proteins); Swiss-Prot (A manually annotated and reviewed protein sequence database); KO (KEGG Ortholog database); and GO (Gene Ontology).

### 4.5. Phylogenetic Analysis

The construction method for the phylogenetic tree was in accordance with a previous study [35]. Briefly, proteins were aligned using the MUSCLE program and the alignment file was used to create a ML phylogenetic tree using the IQ-TREE version 2.0, as described by Minh et al. (2020) [36]. According to the Bayesian Information Criterion (BIC) value, the best-fit model selected was LG + I + G4. The tree file was modified using the Evolview version 2.0 [37].

### 4.6. Gene Cloning and Expression in Yeast

The gene sequence of *ZsLUS* was predicted based on the transcriptomic and genomic data of sour jujube seedlings. The full-length *ZsLUS* cDNA was amplified from the young leaves of a two-year-old sour jujube plant using a Discover-sc Reverse Transcriptase (Vazyme, Nanjing, China) kit following its instructions. The gene-specific amplification primers were 5′-ATG TGG AAG CTG AAG ATA GCA GAA GGA GGG CC-3′ (Forward) and 5′-TTA TGC AAA CAA GAC GCG GCG GCG G-3′ (Reverse). Then, the gene was ligated into the pESC-His vector through a homologous recombination method using a UniClone One Step Seamless Cloning kit (Genesand, Beijing, China). The vector was linearized by double digestion with SalI and XhoI for subsequent recombination. The homologous recombination primers designed to amplify *ZsLUS* gene were 5′-AAT ACG ACT CAC TAT AGG GCC CGG GCG TCG ACA TGT GGA AGC TGA AGA TA-3′ (Forward) and 5′-CTT AGC TAG CCG CGG TAC CAA GCT TAC TCG AGT TAT GCA AAC AAG ACG CG (Reverse). Following the successful construction of the recombinant vector, the resulting plasmid pESC-His-ZsLUS was transformed into *S. cerevisiae* strain WAT11 via the standard lithium acetate transformation protocol. The empty vector (pESC-His) was also transformed into *S. cerevisiae* strain WAT11 as a control. Yeast induction was performed following a protocol established in our laboratory. Briefly, after 48 h of cultivation in a synthetic dropout (SD) liquid medium supplemented with 2% (*w*/*v*) glucose, transgenic yeast cells were harvested by centrifugation and resuspended in fresh SD medium containing 2% (*w*/*v*) galactose to adjust the final OD_600_ to 0.8. The cell suspension was then incubated for an additional 48 h to induce protein expression. Following induction, cells were collected by centrifugation, refluxed with 2 mL of 20% KOH dissolved in 50% ethanol for 5 min, and the target compounds were extracted into hexane. The combined hexane extracts were derivatized with Bis-N,O-(trimethylsilyl) trifluoroacetamide (BSTFA) and pyridine at 65 °C for 45 min before being subjected to GC–MS analysis.

### 4.7. GC-MS Analysis

The GC-MS analysis followed a protocol described by Huang et al. [38] using an Agilent 7890B gas chromatograph coupled to a 5977A mass selective detector (Agilent Technologies, Santa Clara, CA, USA). Separation was carried out on a DB-5MS capillary column (30 m × 0.25 mm i.d., 0.25 μm film thickness). Samples (1 μL) were injected using splitless mode (helium carrier, 1.2 mL/min; injector temperature 250 °C). The column temperature was programmed as follows: 80 °C for 2 min, ramped at 20 °C/min to 290 °C, and held at 290 °C for 20 min. Full scan mass spectra *(m*/*z* 50–600) were acquired, and the target compounds were identified by matching retention times and mass spectra with authentic standards.

## 5. Conclusions

This study demonstrates that ATP treatment modulates the growth and development of sour jujube seedlings, inducing typical phenotypic alterations including inhibited growth, leaf curling and leaf-edge withering. Concurrently, ATP treatment promotes the biosynthesis of triterpenoids. Transcriptomic analysis reveals that core enzyme genes in the mevalonate (MVA) pathway are not uniformly upregulated at the transcriptional level; instead, different isoforms of these enzyme genes exhibit distinct regulatory patterns. The ATP-induced increase in triterpenoid accumulation in sour jujube likely involves an uncharacterized pathway-specific regulatory module or a post-translational modification-dependent regulatory mechanism, which represents a promising direction for further dissection of triterpenoid biosynthesis pathways. In addition, through oxidosqualene cyclase gene analysis and heterologous expression in yeast, a novel sour jujube gene, *ZsLUS*, was identified to encode a bona fide lupeol synthase, which catalyzes the formation of lupeol, a core precursor of lupane-type pentacyclic triterpenoids. These findings provide direct functional evidence for a key enzyme in the lupane-type triterpenoid biosynthesis pathway of sour jujube, filling a gap in the molecular identification of lupeol synthase genes within the *Ziziphus* genus. Collectively, the present work establishes ATP as an effective elicitor for triterpenoid biosynthesis in sour jujube, and provides a functional gene for targeted engineering of lupane-type triterpenoid production. Further studies will focus on the spatiotemporal expression patterns of *ZsLUS* and its upstream transcriptional regulatory mechanism to enable precise, targeted modulation of lupane-type pentacyclic triterpenoid biosynthesis in sour jujube.

## Data Availability

The data that support the findings of this study have been deposited in the China National GeneBank DataBase (CNGBdb) and are publicly available under the accession number CNP0008306.

## Supplementary Materials

The following supporting information can be downloaded at: https://www.mdpi.com/article/10.3390/plants15213367/s1. Table S1: Phenotype and triterpenoid content data of sour jujube seedlings; Table S2: Gene expression values and annotation data; Table S3. Expression data and annotation results for all significant differentially expressed genes; Table S4. Expression changes of genes encoding key enzymes involved in the common triterpenoid biosynthesis pathway; Table S5. Characteristics of the 13 OSC sequences of *Ziziphus jujuba* var. *spinosa*.
